# Precision nursing care of a newborn with congenital absence of skin in both lower extremities: a case report

**DOI:** 10.3389/fped.2026.1825069

**Published:** 2026-05-21

**Authors:** Jiaojiao Liu, Xiuqin Sun, Lamei Zhang, Yan Chen, Xianqun Liu, Yongli Zhu

**Affiliations:** 1 Department of Pediatrics, Affiliated Hospital of North Sichuan Medical College, Nanchong, China; 2 School of Nursing, North Sichuan Medical College, Nanchong, China

**Keywords:** both lower extremities, congenital absence of skin, MDT model, neonate, wound care

## Abstract

This paper summarizes the nursing experience of a neonate with congenital extensive skin defects of both lower extremities. The key nursing strategies included establishing a staged, precision wound-care pathway based on a dynamic and accurate multidisciplinary team (MDT) collaboration mechanism; developing a three-dimensional infection prevention and control nursing model integrating environment, wound, and systemic management; implementing patient-centered, systematic comfort-oriented pain management; and enhancing caregivers' confidence and treatment adherence through structured health education, skills empowerment, and psychological support. After 21 days of systematic treatment and nursing care, the patient's condition stabilized and improved, and the neonate was discharged. Follow-up at two months showed good wound healing. This case provides a referenceable precision nursing model for neonates with congenital extensive skin defects.

## 1 Introduction

Congenital absence of skin (CAS), also known as aplasia cutis congenita (ACC), is a rare developmental disorder characterized by localized or extensive absence of skin at birth. One or more areas may present with congenital defects involving the epidermis, dermal appendages, and even subcutaneous tissue, and the condition may be accompanied by multiple anomalies or systemic malformations (1, 2). The incidence among live births is approximately 1–3 per 10,000 (3). Although the etiology of CAS remains unclear, genetic factors are currently the most widely accepted cause (4, 5). The clinical manifestations of CAS are diverse and complex, presenting as single or multiple lesions that may occur symmetrically or asymmetrically. The condition most commonly affects the scalp (6), but it can also involve other parts of the body, such as the extremities and trunk (7). Congenital CAS involving both lower extremities is extremely rare. At present, there are few domestic reports on nursing experience related to neonates with congenital bilateral lower extremity skin defects. In September 2024, our department admitted a neonate with severe congenital skin defects of both lower extremities. After active treatment and comprehensive nursing care, the patient improved and was discharged. The nursing experience is summarized as follows.

## 2 Clinical data

### 2.1 General information

The patient was a male neonate admitted to the hospital due to “skin defects identified more than 2 h after birth.” He was delivered by cesarean section at a local Maternal and Child Health Hospital on September 17, 2024, with a birth weight of 3,300 g. Apgar scores were 9 at 1 min (1 point deducted for skin color), and 10 at both 5 and 10 min. Physical examination on admission revealed a body temperature of 36.5 °C, heart rate of 138 beats/mina, respiratory rate of 46 breaths/min, and blood pressure of 58/39 mmHg. Extensive skin defects were observed on both lower extremities, measuring 17 × 7 cm on the left lower limb and 8 × 7 cm on the right lower limb. Additional defects were noted on the dorsum of the feet (5 × 3 cm on the left and 4 × 3 cm on the right) and on the plantar surface of the left foot (4 × 3 cm). According to the burn surface area assessment method (8), the total area of skin loss accounted for approximately 15% of the total body surface area. The lesions appeared as exposed red and thin dermal tissue, with no obvious bleeding or exudation. Scattered petechiae were observed on the right side of the back. Laboratory examinations showed a white blood cell count of 22.10 × 10⁹/L, neutrophil percentage of 77.00%, Platelet count of 149*10^9^/L, C-reactive protein level of 2.76 mg/L, and procalcitonin level of 2.302 ng/mL. Stool routine and occult blood testing revealed small fat droplets (++). The patient's father had a history of thigh skin defects. The diagnosis at admission was congenital absence of skin.

### 2.2 Treatment and outcome

**Figure 1:**
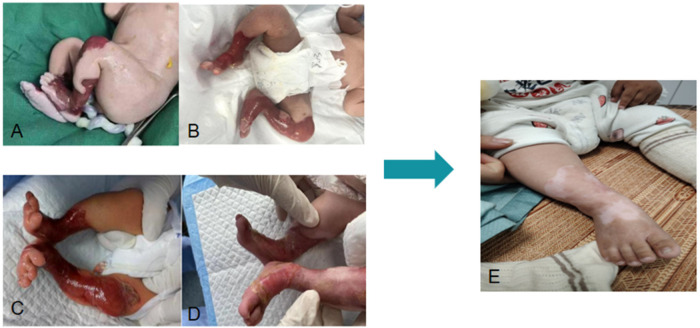
Evolution of wound healing **(A)** At birth; **(B)** At admission; **(C)** Day 3 after admission; **(D)** Day 21 after admission; **(E)** At 2-month follow-up.

On the day of admission, the wounds were cleansed with warm normal saline, followed by the application of Suile® wound ointment combined with recombinant human epidermal growth factor gel. The wounds were then covered with petrolatum gauze and secured with gauze bandages. A multidisciplinary team (MDT) approach was immediately initiated, and emergency consultations were conducted with pediatric surgery, dermatology, plastic and burn surgery, and wound and ostomy specialist nursing teams to jointly develop an individualized treatment and nursing plan for the patient. After multidisciplinary discussion, the wound dressing protocol was adjusted as follows: cleansing with warm normal saline, application of epidermal growth factor gel, Suile® wound ointment combined with a lipid hydrocolloid dressing, and fixation with elastic netting. Dressings were changed once daily. On day 3, based on the patient's condition, topical mupirocin ointment was added, along with intravenous cefotaxime for anti-infective treatment. On day 7, the caregivers strongly requested discharge, and the patient was discharged after signing informed consent and corresponding discharge preparation work. The patient was readmitted on day 8 due to increased wound bleeding compared with the previous condition. Wound exudate was collected for bacterial culture. The wound care regimen was adjusted to cleansing with warm normal saline, irrigation with warm diluted povidone-iodine solution (1:20), followed by application of mupirocin ointment, epidermal growth factor gel, Suile® wound ointment, silver-containing dressings, and fixation with elastic netting. On day 12, the intensity of nursing care was dynamically adjusted, and the dressing change frequency was reduced from once daily to once every other day. The wound care procedure included cleansing with warm normal saline, application of epidermal growth factor gel, Suile® wound ointment combined with a lipid hydrocolloid dressing, and fixation with elastic netting. On day 21, the caregivers again requested discharge, and the patient was discharged after signing informed consent. At that time, the wound area had decreased, exudation was significantly reduced, and a yellow-white membranous layer was observed covering the wound surface. After discharge, the patient received outpatient specialist follow-up and continuous nursing care. The family's request for discharge was closely related to their insufficient understanding of the disease prognosis and elevated anxiety levels. However, through systematic health education and psychological support, there was a significant improvement in family compliance. At the 2-month follow-up, wound evaluation showed scar formation on both lower extremities. The evolution of wound healing is shown in Figure 1.

## 3 Nursing care

### 3.1 A dynamic precision wound care pathway based on multidisciplinary team (MDT) collaboration

#### 3.1.1 Establishment and operation of a dynamic MDT collaboration mechanism

Upon admission, a customized multidisciplinary team (MDT) care model was immediately initiated for the patient. The MDT integrated neonatologists, pediatric surgeons, dermatologists, plastic and burn surgeons, internationally certified wound, ostomy, and continence therapists (WOC nurses), clinical nutritionists, and rehabilitation therapists. A “dual-track parallel, stage-oriented leadership” collaboration mechanism was established. During the acute phase, the neonatologist served as the primary medical coordinator, prioritizing the stabilization of vital signs and internal homeostasis. Concurrently, wound care specialists and pediatric surgeons jointly led early wound bed preparation, performed early surgical debridement, and managed any associated congenital anomalies. During the reparative phase, plastic and burn surgeons, together with rehabilitation therapists, assumed a leading role in collaboratively planning long-term functional and aesthetic reconstruction to maximize preservation of lower limb function. Dermatologists participated throughout the entire treatment process, focusing on etiological investigation and genetic counseling, thereby providing fundamental explanations and guidance for the family. Clinical nutritionists were responsible for calculating nutritional requirements under hypermetabolic conditions and providing professional recommendations regarding feeding strategies and nutritional supplementation. Under the MDT framework, a Primary Nursing management model led by core responsibility nurses is implemented. A senior wound and ostomy specialist nurse serves as the nursing coordinator, responsible for assessment and integration, supervision of plan implementation, and cross-professional communication, ensuring the continuity and consistency of nursing decisions. This collaborative approach facilitates dynamic adjustment of nursing priorities at different treatment stages, ensuring that the most appropriate experts lead decision-making at various disease stages, achieving optimal allocation of medical resources and precise and continuous wound care.

#### 3.1.2 Structured, quantitative, and prospective wound assessment

**Figure 2:**
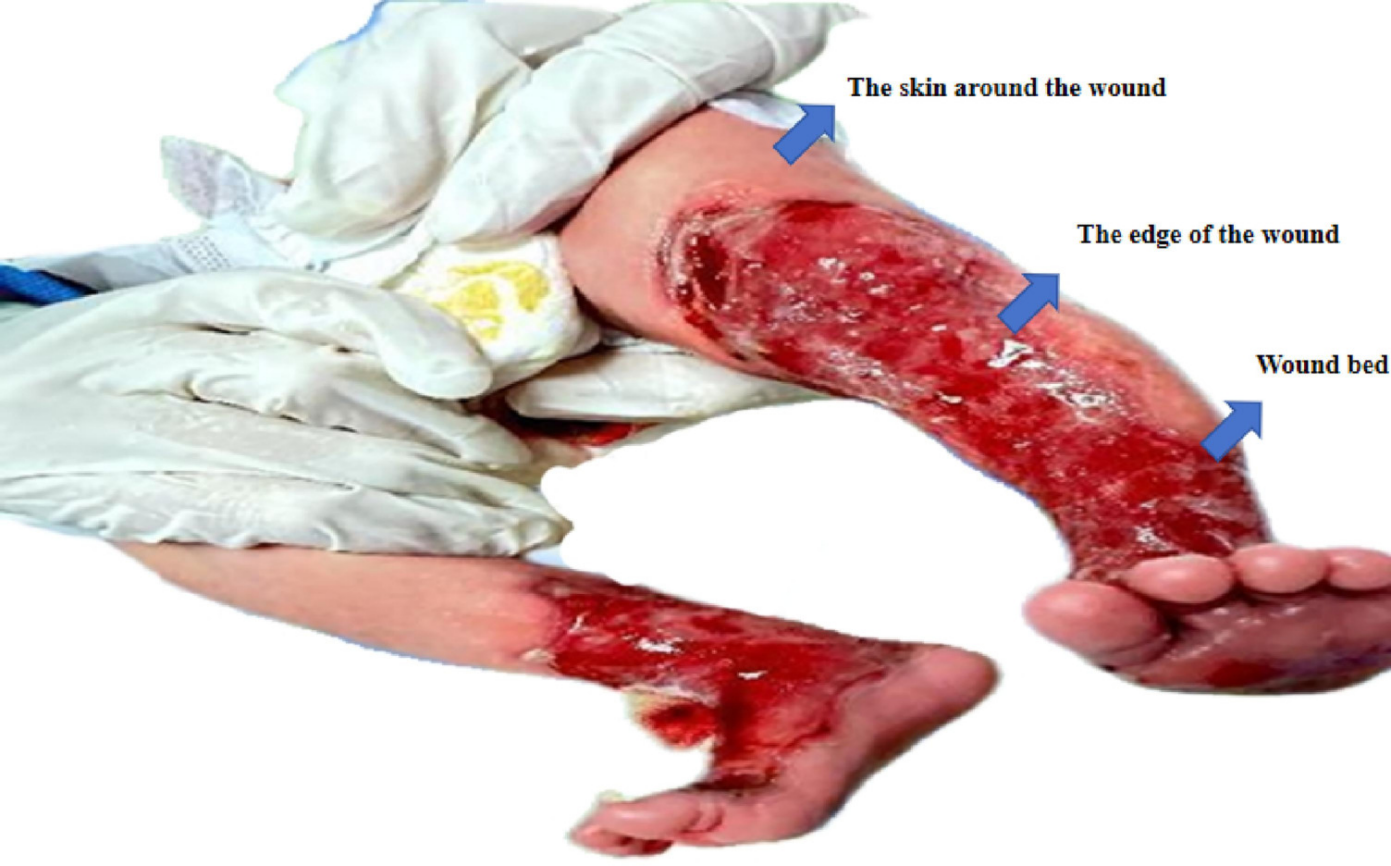
Triangular assessment of the wound surface.

The wound triangle assessment method (9) was introduced for the children's wounds (Figure 2). Based on the characteristics of neonatal skin structure and physiology, adaptive adjustments were made for pediatric applications. The assessment is not only descriptive but also structured and forward-looking. This assessment was not merely descriptive but was structured, quantitative, and prospective in nature. Coordinated by the wound care specialist, the multidisciplinary team (MDT) comprehensively and systematically evaluated the patient's condition from local wound characteristics to overall systemic status. (1) Local wound assessment: At admission, the skin defects involved the epidermis and partial dermal layers, with clearly defined margins. Red, thin dermal tissue was exposed at the defect sites, with no obvious exudation or fresh bleeding observed. The extent of skin loss on both lower extremities was as follows: 17 × 7 cm on the left lower limb, 8 × 7 cm on the right lower limb, 5 × 3 cm on the left dorsal foot, 4 × 3 cm on the right dorsal foot, and 4 × 3 cm on the left plantar surface. No maceration or dehydration was observed at the wound edges, and the surrounding skin was smooth and intact without abnormalities. (2) Systemic condition: Systemic evaluation was closely integrated with nutritional indicators assessed by the nutritionist, including daily caloric intake and serum prealbumin levels, as well as infection-related markers such as C-reactive protein (CRP) and procalcitonin, and pain assessment scores. (3) Healing environment and risk factors: Assessment of the healing environment included evaluation of body positioning, friction-related risks, and the level of family support. This structured assessment approach provided objective and comparable data to support precision interventions.While facilitating infection control, wound management, and optimization of the healing environment, it also enabled early identification of delayed healing or potential infection risks.

#### 3.1.3 Precision intervention strategies based on the physiological stages of wound healing

In accordance with the physiological stages of wound healing—namely the inflammatory phase, proliferative phase, and remodeling phase—the wound care regimen for the patient's skin defects was dynamically adjusted under evidence-based guidance and multidisciplinary team (MDT) collaboration. Inflammatory/debridement phase (Days 1–11): Previous studies have indicated that the early stage of wound healing is characterized by abundant exudate and fragile granulation tissue, with nursing priorities focused on infection prevention and minimization of mechanical injury (10). Warm normal saline was used for pulsatile irrigation to effectively remove contaminants while minimizing mechanical trauma. Normal saline is non-cytotoxic to healthy cells, maintains osmotic stability, and reduces local irritation (11).

The Wound Hygiene concept proposed by the International Wound Infection Institute (IWII) was integrated into clinical practice, shifting infection management from passive response to proactive prevention. This approach included: ① Cleanse: circular irrigation with warm normal saline from the center outward to reduce microbial load; ② Debride: gentle debridement primarily through autolytic methods, using hydrogel or enzymatic debriding agents to promote endogenous enzymatic breakdown; ③ Refreshed cleanse: neutralization of wound pH and removal of substances that might interfere with subsequent dressing adhesion or activity; ④ Edge and periwound protection: maintenance of a moist but non-exudative wound microenvironment, with the use of non-adhesive dressings and tubular elastic netting for fixation.

These steps were standardized into a reproducible physical wound hygiene protocol. Based on exudate assessment and evidence-based support, dressing selection was dynamically optimized. Highly absorbent lipid hydrocolloid dressings or silver-containing dressings were applied. Lipid hydrocolloid dressings effectively absorbed large volumes of exudate while maintaining an optimal moist environment, whereas silver-containing dressings provided broad-spectrum antimicrobial protection, thereby reducing the intensity and frequency of systemic antibiotic use (12). In addition, Suile® wound ointment containing nanosilver was combined with recombinant human epidermal growth factor gel to promote keratinocyte migration and proliferation and accelerate epithelialization (13). Proliferative phase (Day 12 to discharge): The primary goals during this phase were to promote granulation tissue formation and epithelialization. Once exudation decreased and healthy granulation tissue appeared, the intervention strategy was promptly adjusted. Rehabilitation therapists were concurrently involved to guide passive joint range-of-motion exercises within non-invasive limits to prevent joint contractures, reflecting the synchronization of functional rehabilitation and wound healing. Remodeling phase (post-discharge follow-up): The focus during this phase was scar management and maximization of functional outcomes. During specialist outpatient follow-up, caregivers were instructed in scar pressure therapy (using customized elastic garments), application of silicone-based products, and moisturizing massage, along with continued rehabilitation exercises.

Following 21 days of inpatient interventions and 2 months of continuous post-discharge nursing care, the patient's wounds healed successfully, ultimately forming soft and flat scars, with good preservation of lower limb joint mobility. This practice has demonstrated the potential value of staged precise care in optimizing the healing outcome of neonatal wounds.

### 3.2 A three-dimensional infection prevention and control strategy integrating environment, wound, and systemic management

A three-dimensional protective framework integrating environmental control, local wound management, and systemic monitoring was established to minimize infection risk. The initiation of anti-infective therapy was based on the severe disruption of the patient's skin barrier, immature immune function, and laboratory indicators suggesting a potential risk of infection, with treatment dynamically adjusted under close monitoring. This strategy required protective isolation, enhanced basic nursing care, meticulous wound management, and a rigorous monitoring and early-warning system. Specific measures were implemented as follows: ① The neonate was placed in a single-room laminar airflow ward under protective isolation. Medical staff wore masks, caps, isolation gowns, and gloves, strictly adhered to hand hygiene protocols, assigned dedicated caregivers, and restricted unnecessary personnel movement. ② All surfaces in the ward, including the incubator, monitors, and infusion pumps, were disinfected twice daily using a chlorine-containing disinfectant solution at a concentration of 1,000 mg/L. ③ Umbilical care was performed twice daily to maintain cleanliness and dryness. General skin hygiene was ensured, with bedside sponge bathing once daily and oral care twice daily. ④ Strict aseptic technique was enforced during all wound care procedures. Hand hygiene was repeated before dressing changes, and sterile gloves were worn throughout. ⑤ Dressing change frequency was optimized to maintain a moist wound bed without fluid accumulation while protecting the surrounding skin from excessive moisture. Based on exudate levels, dressings were changed once daily during the first 11 days and subsequently adjusted to once every other day. ⑥ Wound exudate was routinely collected for bacterial culture. Laboratory markers, including complete blood count, C-reactive protein (CRP), and procalcitonin, were dynamically monitored, and anti-infective therapy was adjusted accordingly (14). Topical mupirocin ointment and intravenous cefotaxime were administered as indicated. ⑦ Close observation of wound characteristics—including size, depth, color, odor, amount and nature of exudate (color and viscosity), and periwound tissue status (erythema, swelling, and temperature)—was conducted to establish an infection early-warning assessment system. In this case, no local or systemic infection alarms were triggered, and wound healing progressed as expected. It demonstrates the effectiveness and necessity of the “trinity” protective network in managing the protection of immunocompromised newborns from fatal infections, providing practical reference ideas for the infection control management of similar high-risk children.

### 3.3 Patient-centered systematic comfort-oriented pain management

Pain was routinely managed as the “fifth vital sign,” following the principles of preventive, multimodal, and individualized analgesia. A closed-loop comfort-oriented management framework of “assessment–prevention–intervention–reassessment” was established to ensure systematic and continuous pain control (15). Pain management began with a comprehensive and dynamic assessment. Routine baseline evaluations were conducted daily to monitor background pain levels. Predictive assessments were performed 15 min before all planned procedures (such as debridement, dressing changes, and position adjustments) to guide the intensity of preemptive analgesia. In addition, procedural assessments were carried out at peak pain moments during interventions, as well as 10 and 30 min after completion, to evaluate the effectiveness of implemented measures. All assessment results and corresponding interventions were systematically documented in nursing records. Appropriate analgesic and comfort measures were selected based on pain scores. Specific nursing interventions included the following: ① Environmental and basic comfort management: Nursing procedures were clustered to reduce repeated stimulation, and a quiet, low-light, low-irritation environment was maintained. ② Procedural pain prevention: During debridement and dressing changes, all actions were performed gently. Dressings were routinely moistened with warm normal saline before removal, significantly reducing mechanical pain caused by adhesion. When the Neonatal Infant Pain Scale (NIPS) score was ≥3, saline soaking time was extended accordingly. ③ Positioning comfort support: For lower extremity wounds, an innovative method was applied by placing a partially inflated medical rubber glove pad beneath the calf. This created a soft, adjustable support surface that effectively dispersed pressure and reduced positional pain as well as wound friction. ④ Non-pharmacological interventions: Prior to potentially painful procedures, if the NIPS score was ≥3, a pacifier dipped in glucose solution was provided in advance for non-nutritive sucking, serving as a foundational analgesic measure. ⑤ Pharmacological analgesic escalation: When the NIPS score was ≥4 (moderate or greater pain) and non-pharmacological interventions were insufficient, stepwise pharmacological analgesia was administered strictly according to medical orders. Timely reassessments were conducted after medication to ensure adequate pain relief. In this case, through systematic dynamic assessment and intervention, the patient's pain was effectively controlled. The NIPS score decreased from 4 points (moderate pain) at admission to 3 points (mild pain) by day 9, and further stabilized at 2 points (no pain or mild discomfort) by day 16. This downward trend highlights the important role of systematic pain management in improving the quality of care and humanistic support for critically ill neonates, while objectively confirming the effectiveness and continuity of the implemented pain management strategy.

### 3.4 Empowering the family: structured health education and psychological support

This case adopts Family-Centered Care (FCC) as an important nursing management strategy. Through systematic assessment, tiered education and continuous support, family members are transformed from passive recipients of care to co-decision-makers and executors of care. When faced with the rare defect wounds of the patients, the widespread severe psychological crises and lack of knowledge among the families led to the implementation of a four-pronged family intervention program of “assessment - education - empowerment - support”. Initial assessment and empathic intervention, at the early stage of hospitalization, the Self-Rating Anxiety Scale (SAS) was administered. The results showed that the SAS standard score of the family members was 65, indicating moderate anxiety. Due to the prolonged treatment course and high uncertainty regarding prognosis, the caregivers initially doubted the likelihood of successful recovery. This raised concerns about treatment adherence, and at one point, they strongly requested discharge against medical advice. In accordance with the nursing philosophy of being “patient-centered with family-based support” (16), systematic health education, psychological counseling, and skill empowerment were provided to enhance caregivers’ understanding and confidence. Specific interventions included the following: ① Empathic communication: Upon admission, healthcare professionals used gentle and empathic language to acknowledge and understand the caregivers’ sadness and anxiety, providing emotional comfort and psychological support. ② Structured information delivery: Disease characteristics, treatment plans, and prognosis were explained in a stepwise and progressive manner. This approach transformed the “fear of the unknown” into a “manageable challenge,” helping caregivers rebuild their cognitive framework. ③ Instilling hope and confidence: Successful recovery cases were shared to strengthen caregivers’ hope and confidence. Families were encouraged to actively participate in nursing care, with emphasis on their critical role in the treatment process. ④ Skill demonstration and empowerment: Once the patient's condition stabilized, caregivers were trained using a “demonstration–collaboration–independence” model. Parents gradually learned safe wound observation and basic positioning care, shifting from passive “bystanders” to active “collaborators,” thereby improving their sense of control and self-efficacy. ⑤ Establishing a continuity support network: A discharge guidance manual was developed, and an exclusive WeChat follow-up group was established. Continuous support was provided for post-discharge wound management, progressive limb function and activity guidance, and ongoing psychological and social support. Core nursing team members offered regular consultations, emotional counseling, and rehabilitation guidance, ensuring seamless transition from hospital to home and delivering sustained care.

Through these interventions, caregivers demonstrated positive psychological and behavioral changes. Their attitude shifted from initial doubt and withdrawal to proactive inquiry and cooperation. At discharge reassessment, the caregivers’ SAS standard score decreased from 65 to 52, indicating a reduction of anxiety to the mild range.

## 4 Conclusion

The successful management of this neonate with extensive congenital skin defects involving both lower extremities highlights the advantages of an integrated care model driven by an innovative multidisciplinary team (MDT) collaboration mechanism. This model incorporated precision wound care, a three-dimensional infection prevention and control strategy, systematic comfort-oriented pain management, and a family empowerment framework, achieving favorable clinical outcomes. In the future, we can actively explore new types of biological dressings such as Janus hydrogels (17), and study cutting-edge technologies like bioactive polymer dots and laser technology (18) to provide theoretical support for nurses to participate in the selection and follow-up management of new wound materials; and utilize digital tools such as 3D wound imaging and remote monitoring to achieve more accurate assessment and follow-up.

## Data Availability

The original contributions presented in the study are included in the article/Supplementary Material, further inquiries can be directed to the corresponding author.
