# Supplementary material for: Precision nursing care of a newborn with congenital absence of skin in both lower extremities: a case report
Source: Front Pediatr. 2026 May 21;14:1825069. doi: 10.3389/fped.2026.1825069 (PMC13233448; doi:10.3389/fped.2026.1825069)
Supplement: Supplementary file 1 [file Table1.docx]

Supplementary Material

# Supplementary Tables

Table 1. Serial laboratory findings

| Date | WBC (×10⁹/L) | Neutrophils (%) | CRP (mg/L) | Procalcitonin (ng/mL) | Wound culture |
| --- | --- | --- | --- | --- | --- |
| 2024.9.17 | 22.10 | 77 | 2.76 | 2.302 | Negative for CRE |
| 2024.9.20 | 7.32 | 50.3 | 5.42 | 1.232 | / |
| 2024.9.24 | 7.80 | 17.60 | 37.8 | 0.31 | Negative for CRE |
| 2024.9.26 | 8.78 | 50.40 | 11.90 | 0.220 | Blood cultures ① and ② were negative |
| 2024.10.2 | 7.39 | 36.6 | 3.26 | 0.117 | / |

# *CRE:Enterobacteriaceae resistant to carbapenems;①Anaerobic culture for 5 days resulted in sterile growth;②Aerobic culture for 5 days resulted in sterile growth.
